# Postoperative hospitalization time in laparoscopic and laparotomic Roux-En-Y gastric bypass: a retrospective observational cohort study

**DOI:** 10.1590/0102-672020260000043e1972

**Published:** 2026-09-18

**Authors:** Tomer Lederman BOOKOBZA, Gabriel Scaramuzzi SEFERIAN, Giovanna Pedrazzoli RODRIGUES, Wilson Rodrigues FREITAS, Osvaldo Antônio Castro PRADO, Luiz Vicente BERTI, Elias Jirjoss ILIAS, Patrícia Colombo SOUZA, Paulo KASSAB

**Affiliations:** 1 Santa Casa de São Paulo, Faculty of Medical Sciences – São Paulo (SP), Brazil.; 2 Santa Casa de São Paulo, Faculty of Medical Sciences, Department of Surgery – São Paulo (SP), Brazil.; 3 Universidade Santo Amaro – São Paulo (SP), Brazil.

**Keywords:** Obesity, Bariatric Surgery, Laparotomy, Laparoscopy, Length of Stay, Obesidade, Cirurgia Bariátrica, Laparotomia, Laparoscopia, Tempo de Internação

## Abstract

**Background:**

Obesity is a growing problem both in Brazil and worldwide, and bariatric surgery is a well-established method for reducing weight and, consequently, reducing the risk of death attributed to cardiovascular and metabolic problems. Roux-en-Y gastric bypass is currently the most popular surgical technique in Brazil, performed via either laparotomy or laparoscopy.

**Aims:**

To compare the length of hospital stay after Roux-en-Y gastric bypass performed via laparoscopic and laparotomic approaches.

**Methods:**

An observational study using retrospective data from medical records, comparing patients who underwent Roux-en-Y gastric bypass by laparoscopy and patients operated on by laparotomy, with similar comorbidities.

**Results:**

The sample consisted of 64 patients who underwent surgery: 31 via laparoscopy and 33 via laparotomy, of whom 84.4% were female, with a mean age of 44.1 (SD=10.1). 15.6% were male, with a mean age of 40.2 (SD=13.3). Among the four variables evaluated (sex, age, length of hospital stay, and type of surgery), the only statistically significant difference found was in the length of hospital stay according to each type of surgery, with patients undergoing Roux-en-Y gastroplasty via laparoscopic approach remaining hospitalized for a mean of 2.45 days (SD=1.12), while those undergoing the same surgery via laparotomic approach remained hospitalized for a mean of 3.42 days (SD=1.17).

**Conclusions:**

The findings of this study indicate a numerically and statistically significant difference in length of hospital stay between both surgical access routes for bariatric surgery, with a shorter stay observed in the laparoscopic approach. This information is relevant, as it has financial impact by reducing bed occupancy time and the costs arising from hospitalization.

## ARTICLE HIGHLIGHTS

Obesity is a growing problem both in Brazil and worldwide. Its rise is associated with an increased risk of death from various causes, such as cardiovascular disease, diabetes, and cancer, as well as a significant decline in quality of life and life expectancy.

Gastric bypass, a mixed procedure also known as Roux-en-Y gastroplasty, has become the most popular surgical technique in Brazil (accounting for 93.7% of all procedures).

Given the increasing indication for bariatric surgery, it is important to identify the safest approach, associated with lower morbidity and mortality and resulting in better patient outcomes.

The findings of this study indicate a numerically and statistically significant difference in length of hospital stay between both surgical access routes for bariatric surgery, with a shorter stay observed in the laparoscopic approach.

## Summary

**CENTRAL MESSAGE:**

Obesity is a growing problem both in Brazil and worldwide. Bariatric surgery is a well-established method for reducing weight and, consequently, reducing the risk of death attributed to cardiovascular and metabolic problems. Gastric bypass, a mixed procedure also known as Roux-en-Y gastroplasty, is currently the most popular surgical technique in Brazil. In recent years the laparoscopic approach has become widely used for this procedure.

**PERSPECTIVES:**

The findings of this study indicate a numerically and statistically significant difference in length of hospital stay between both surgical access routes for bariatric surgery, with a shorter stay observed in the laparoscopic approach. This information is relevant to the medical field because, in addition to improving the quality of life of the patients and their overall hospital experience, it also has a financial impact by reducing bed occupancy time and the costs arising from hospitalization.

## VISUAL ABSTRACT

**Figure:**
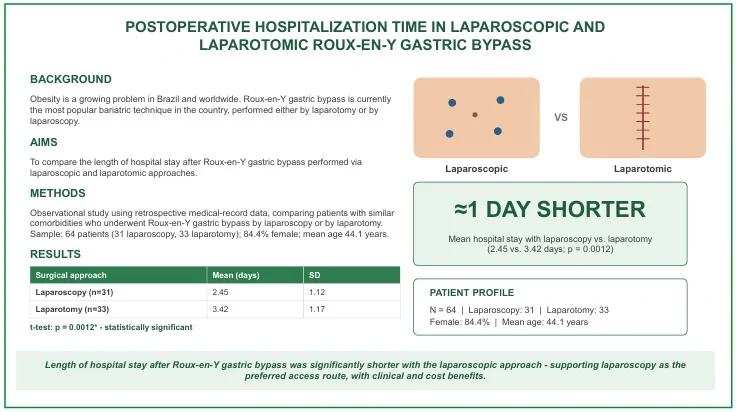

## RESUMO

**Racional:**

A obesidade é um problema crescente tanto no Brasil quanto mundialmente, e a cirurgia bariátrica é um método bem estabelecido para a redução de peso e, consequentemente, para a diminuição do risco de morte associado a problemas cardiovasculares e metabólicos. O bypass gástrico em Y de Roux é atualmente a técnica cirúrgica mais utilizada no Brasil, realizada por via laparotômica ou laparoscópica.

**Objetivos:**

Comparar o tempo de permanência hospitalar após o bypass gástrico em Y de Roux realizado pelas vias laparoscópica e laparotômica.

**Métodos:**

Estudo observacional utilizando dados retrospectivos de prontuários, comparando pacientes submetidos ao bypass gástrico em Y de Roux por laparoscopia e pacientes operados por laparotomia, com comorbidades semelhantes.

**Resultados:**

A amostra consistiu em 64 pacientes operados: 31 por laparoscopia e 33 por laparotomia, sendo que 84,4% eram do sexo feminino, com idade média de 44,1 anos (DP=10,1), e 15,6% eram do sexo masculino, com idade média de 40,2 anos (DP=13,3). Entre as quatro variáveis analisadas (sexo, idade, tempo de permanência hospitalar e tipo de cirurgia), a única diferença estatisticamente significativa encontrada foi no tempo de permanência hospitalar de acordo com o tipo de cirurgia: pacientes submetidos à gastroplastia em Y de Roux por via laparoscópica permaneceram internados por uma média de 2,45 dias (DP=1,12), enquanto aqueles submetidos à mesma cirurgia por via laparotômica permaneceram internados por uma média de 3,42 dias (DP=1,17).

**Conclusões:**

Os achados deste estudo indicam uma diferença numérica e estatisticamente significativa no tempo de permanência hospitalar entre as duas vias de acesso para a cirurgia bariátrica, observando-se um tempo menor na abordagem laparoscópica. Essa informação é relevante, pois gera impacto financeiro ao reduzir o tempo de ocupação do leito e os custos decorrentes da hospitalização.

## INTRODUCTION

 Obesity is a growing problem both in Brazil and worldwide^14^. Its rise is associated with an increased risk of death from various causes, such as cardiovascular disease, diabetes, and cancer^1 ,10,12^, as well as a significant decline in quality of life and life expectancy^2^. Bariatric surgery is a well-established method for reducing weight and, consequently, reducing the risk of death attributed to cardiovascular and metabolic problems^3,25^ . 

 Although its earliest records date back to the 10th century, it was only in the 20th century that bariatric surgery became widespread in medical practice^9^. In 1952, the first surgery for obesity, a small bowel resection, was performed. In the 1970s, restrictive techniques, such as vertical gastrectomy, became popular, mainly due to the publications of Faria et al.^9^. The end of the 1970s and the beginning of the 1980s saw the emergence of malabsorptive techniques (such as the duodenal switch) and other mixed approaches^21^. Gastric bypass, a mixed procedure also known as Roux-en-Y gastroplasty, has become the most popular surgical technique in Brazil (accounting for 93.7% of all procedures)^4,12^ . 

 Nevertheless, it was only near the end of the last century, with the recognition of obesity as an epidemic, that greater efforts were invested in improving and diversifying surgical techniques^9^. In 1994, the first laparoscopic Roux-en-Y bypass was performed by Faria et al.^9^, forever changing the history of bariatric surgery. Subsequently, literature underwent a process of continuous expansion, documenting a decrease in morbidity while maintaining promising results and safety^8,21,23^. Currently, laparoscopic bariatric surgery has become the most popular approach due to its minimally invasive nature. 

 When both surgical approaches are compared, authors report that the laparoscopic approach is associated with a lower incidence of postoperative infection and a lower incidence of incisional hernia^4,16,25^. In addition, the laparoscopic approach is associated with lower postoperative pain and a shorter recovery time, resulting in an earlier return to work and daily activities^4,11,16^. 

 However, some facts remain controversial in literature. Mortality after bariatric surgery is a relevant factor in the analysis of the procedure; while some studies show no evidence of significant differences^24 ,25^, others indicate lower mortality with the laparoscopic approach^4,11^. 

 Other factors associated with the procedure remain debatable and lack a high level of scientific evidence, although they point toward the laparoscopic technique as favorable, such as the incidence of postoperative pulmonary complications^7,11,25^, reoperation rate^11,16 ,25^, and weight loss^16^. 

 Interestingly, even though laparoscopic bariatric surgery is associated in the literature with a shorter recovery time and lower complication rates^6,19,24^, its role in reducing costs compared with the laparotomic approach is still under discussion. 

 Given the increasing indication for bariatric surgery, it is important to identify the safest approach, associated with lower morbidity and mortality, therefore resulting in better patient outcomes. 

## METHODS

 Data from patients who underwent Roux-en-Y gastric bypass by laparoscopy were compared with those who underwent the same procedure via laparotomy, through retrospective data collection from medical records of the Obesity Unit of the Department of Surgery of the Santa Casa de São Paulo.

- Inclusion criteria: Patients with morbid obesity, with comparable BMI and comorbidities, of both sexes, aged over 18 years and up to a maximum of 70 years;
- Exclusion criteria: patients above 70 years of age.

All patients underwent outpatient follow-up, preoperative examinations, were at minimal risk for surgery using either technique, and agreed to the proposed surgical indication. 

 Patients were operated on by the same team of surgeons from the Service, following identical pre- and postoperative care protocols in both groups. 

### Statistical analysis

 Parametric t-tests, the chi-square test, and analysis of variance (Mann-Whitney test) were used to compare both study groups, with a significance level of 5%. The study was approved by the Institution’s Research Ethics Committee (CAAE No. 5479). 

## RESULTS

 The variables used in this study, based on the data collected, were patient sex, age, length of hospital stay, and surgical technique. 

 The sample comprised 64 patients, of whom 31 underwent Roux-en-Y bypass by laparoscopy and 33 underwent the same operation by laparotomy. The vast majority of patients were female (84.4%), with a mean age of 44.1 years. Meanwhile, the 10 men who participated in this study had a mean age of 40.2 years. 

 The results and comparisons of the variables analyzed between the two groups are presented in Tables 1-5 [Table T2] [Table T3] [Table T4] [Table T5]. 

**Table 1:** Age of patients who underwent bariatric surgery, regardless of surgical technique, by sex.

Female	Male
N=54	N=10
Minimum-Maximum= 25–70 years	Minimum-Maximum= 22–65 years
Mean=44.12 years±10.1	Mean=40.2 years±13.3
Median=44.5 years	Median=39.0 years
Variance=1.02	Variance=1.04

Mann-Whitney test: p=0.1112 (one-tailed); p=0.2223 (two-tailed). Variance=114.7692.

**Table 2 T2:** Age of the patients who underwent bariatric surgery, according to surgical technique.

Laparoscopy	Laparotomy
N=31	N=33
Minimum-Maximum= 22–65 years	Minimum-Maximum= 25–70 years
Mean=43.19 years±11.7	Mean=43.8 years±9.8
Median=44 years	Median=44 years
Variance=1.02	Variance=1.04

Variance=116.7701; Mann-Whitney test: p=0.8089 (two-tailed).

**Table 3 T3:** Surgical technique of patients who underwent bariatric surgery, by sex.

Sex	Laparoscopy (N/%)	Laparotomy (N/%)	Total (N/%)
Female	26 (48.2)	28 (51.8)	54 (84.4)
Male	5 (50.0)	5 (50.0)	10 (15.6)
Total	31 (48.4)	33 (51.6)	64 (100.0)

χ^2^ test: p=0.9143.

**Table 4 T4:** Length of hospital stay of patients who underwent bariatric surgery, by sex.

Female	Male
N=54	N=10
Minimum-Maximum=2–4 days	Minimum-Maximum=1–9 days
Mean=2.79 days ± 0.76	Mean=3.8 days ± 2.52
Median=3 days	Median=3 days
Variance=1.02	Variance=1.04

Variance=0.6507; Mann-Whitney test: p=0.4765 (two-tailed).

**Table 5 T5:** Length of hospital stay of patients who underwent bariatric surgery, according to the surgical technique used (Laparoscopy or open).

Laparoscopy	Laparotomy
N= 31	N=33
Minimum-Maximum=1–7 days	Minimum-Maximum=2–9 days
Mean=2.45 days±1.12	Mean=3.42 days±1.17
Median=2 days	Median=3 days
Variance=1.37	Variance=1.25

t-test: p=0.0012* (two-tailed); Variance=1.3184.

 Only one patient among those operated on by the laparoscopic approach presented a postoperative complication: a 58-year-old male patient with type III obesity, type 2 diabetes mellitus, chronic kidney disease, and three previous acute myocardial infarctions, who developed heart failure requiring seven days of hospitalization. No deaths were recorded in this case series. 

 The results showed that the male and female groups had a similar age range, and there was no statistically significant difference between age and sex (Table 1) (p>0.05). 

 There was no statistically significant difference in age when comparing the surgical techniques performed and patient sex (Table 2) (p>0.05). Likewise, no statistically significant difference was found between the surgical approach used and patient sex (Table 3) (p>0.05). 

 When comparing length of hospital stay and patient sex, no statistically significant difference was found (Table 4) (p>0.05). 

 Finally, when comparing length of hospital stay and the surgical approach used, a statistically significant difference was recorded (Table 5) (p<0.05). 

## DISCUSSION

 The variation in length of hospital stay in this study was statistically significant according to the surgical approach used to perform the procedure. However, the literature emphasizes that other important factors must be considered in determining the total length of hospital stay of patients undergoing bariatric surgery. These include comorbidities such as diabetes, hypertension, and/or other diseases, as well as surgical complications and postoperative dietary tolerance^15,18^ . 

 Even accounting for the patient who developed a postoperative complication, the mean length of hospital stay for the laparoscopic method remained one day shorter compared with the open approach, and this difference was both numerically and statistically significant (Table 5). 

 Therefore, in the present study, considering that all patients received similar postoperative care, including dietary reintroduction, and that a postoperative complication occurred in only one case, it is reasonable to conclude that the type of surgery was, in fact, the main determinant of the number of days patients remained hospitalized postoperatively. 

 Among the four variables studied — sex, age, length of hospital stay, and type of surgery — only the latter two were statistically related. This finding is important as it allows us to conclude, with a reasonable degree of certainty, that a longer length of hospital stay cannot be attributed to age (Table 2) and, consequently, to age-related complications^19^. The same conclusion can be drawn regarding patient sex (Tables 3 and 4). 

 Furthermore, it is of great importance to consider the impact of this analysis on public healthcare spending. Turri et al.^23^ reported that, among 318 patients who underwent bariatric surgery at the University Hospital of the Universidade de São Paulo and the Hospital Geral de Suzano (SP) between 2017 and 2018, laparoscopic surgeries had a significantly lower economic impact. Procedures performed via an open approach — namely vertical gastrectomy, adjustable gastric banding (AGB), and Roux-en-Y gastric bypass (RYGB) — had hospitalization costs of US$13,861.88, US$7,284.31, and US$9,244.69, respectively, whereas the same procedures performed by laparoscopy had hospitalization costs of US$4,860.69, US$4,100.72, and US$8,495.18, in the same order^ 22,23^. The hypothesis that laparoscopic surgery would reduce costs is not new: in 1995, researchers in Milwaukee reached the same conclusion, although they still disagreed on the advantages of this procedure regarding weight loss and outcomes^7^. 

 Finally, the advent of anti-obesity medications puts bariatric surgery to the test. After all, should patients not be able to choose the option that, weighing factors such as cost, effectiveness, and comfort, works best for them? Recent studies have shown the validity of pharmacological treatment^ 13,20^, although its cost may hinder adherence^17^. Nevertheless, in 2023 the number of bariatric surgeries increased by 22.9% compared with 2019, i.e., the year before the onset of the Covid-19 pandemic^5^. 

 Thus, the continuous pursuit of knowledge, aiming to offer patients the best possible quality of life and to optimize hospital services, should not only be maintained but encouraged. 

## CONCLUSION

 The findings of this study indicate a numerically and statistically significant difference in length of hospital stay between the two surgical access routes for bariatric surgery, with a shorter stay observed in the laparoscopic approach. This information is relevant to the medical field because, in addition to improving patients’ quality of life and their overall hospital experience, it also offers financial benefits by reducing bed occupancy time and the costs arising from hospitalization. 

## Data Availability

The datasets generated and/or analyzed during the current study are available from the corresponding author upon reasonable request.
